# Engineered skin microbiome reduces mosquito attraction to mice

**DOI:** 10.1093/pnasnexus/pgae267

**Published:** 2024-07-30

**Authors:** Feng Liu, Iliano V Coutinho-Abreu, Robyn Raban, Tam Thuy Dan Nguyen, Alejandra R Dimas, Joseph A Merriman, Omar S Akbari

**Affiliations:** Department of Cell and Developmental Biology, School of Biological Sciences, University of California, San Diego, 9500 Gilman Drive, La Jolla, CA 92093, USA; Department of Cell and Developmental Biology, School of Biological Sciences, University of California, San Diego, 9500 Gilman Drive, La Jolla, CA 92093, USA; Department of Cell and Developmental Biology, School of Biological Sciences, University of California, San Diego, 9500 Gilman Drive, La Jolla, CA 92093, USA; Sarafan ChEM-H, Stanford University, 290 Jane Stanford Way, Stanford, CA 94305, USA; Microbiome Therapies Initiative (MITI), Stanford University, 3165 Porter Drive, Palo Alto, CA 94305, USA; Sarafan ChEM-H, Stanford University, 290 Jane Stanford Way, Stanford, CA 94305, USA; Microbiome Therapies Initiative (MITI), Stanford University, 3165 Porter Drive, Palo Alto, CA 94305, USA; Sarafan ChEM-H, Stanford University, 290 Jane Stanford Way, Stanford, CA 94305, USA; Microbiome Therapies Initiative (MITI), Stanford University, 3165 Porter Drive, Palo Alto, CA 94305, USA; Department of Cell and Developmental Biology, School of Biological Sciences, University of California, San Diego, 9500 Gilman Drive, La Jolla, CA 92093, USA

**Keywords:** engineered human skin microbiota, *Staphylococcus epidermidis*, *Corynebacterium amycolatum*, lactic acid, mosquito repellency

## Abstract

The skin microbiome plays a pivotal role in the production of attractive cues detected by mosquitoes. Here, we leveraged recent advances in genetic engineering to significantly reduce the production of L-(+)-lactic acid as a strategy to reduce mosquito attraction to the highly prominent skin commensals *Staphylococcus epidermidis* and *Corynebacterium amycolatum.* Engraftment of these engineered bacteria onto the skin of mice reduced mosquito attraction and feeding for up to 11 uninterrupted days, which is considerably longer than the several hours of protection conferred by the leading chemical repellent N,N-diethyl-meta-toluamide. Taken together, our findings demonstrate engineering the skin microbiome to reduce attractive volatiles represents an innovative untapped strategy to reduce vector attraction, preventing bites, and pathogen transmission. These findings set the stage for new classes of long-lasting microbiome-based repellent products.

**Significance Statement**

This study demonstrates that two genetically engineered human skin bacteria turn a mammal host less attractive to mosquito bites for over 11 days. This time span is considerably much longer than the 4–8-hour protection provided by the gold-standard synthetic repellent N,N-diethyl-meta-toluamide (DEET). As the human scent is derived from the metabolism of the human skin microbiota, knocking out the synthesis of key mosquito attractants paves the way for the development of a skin therapy that provides a more permanent protection against diseases transmitted by mosquitoes, such as dengue fever, Zika, and malaria.

## Introduction

Mosquitoes are responsible for the transmission of a variety of deadly human pathogens, including malaria, West Nile, dengue, yellow fever, and Zika viruses. In 2022, about 250 million cases of human malaria occurred worldwide (1). While it is still considered to be underestimated, the annual dengue burden accounts for 400 million cases and 22,000 deaths worldwide each year (2). Current topical repellents are potent inhibitors of mosquito attraction (>90% reduction using DEET) (3). However, an important limitation of these repellents is their very short window of protection (4), placing individuals at risk of exposure.

Female mosquitoes ingest host blood required for egg development, through which the pathogens are acquired and transmitted by mosquitoes to humans (5, 6). Mosquitoes rely on their acute olfactory system to detect volatiles, including CO_2_, L-(+)-lactic acid, and other specific odors to locate their hosts (7, 8). Particularly, volatiles emanating from vertebrate skin play essential roles in mosquito host seeking. Indeed, CO_2_ is considered a synergist of skin volatiles, causing stronger behavioral responses in host-seeking mosquitoes than the volatiles alone (9–11). Volatile compounds inform the mosquito about the quality (12) as well as identity (13) of the host, being responsible for the host specificity of a range of mosquitoes. While several host-derived compounds affecting mosquito host seeking have been described, L-(+)-lactic acid remains one of the most prominent mosquito attractive volatiles from human emanation, synergizing with CO_2_ in both laboratory and field applications (9, 14). It was only recently discovered that many of these compounds, including L-(+)-lactic acid, are of bacterial origin (15, 16).

*Staphylococci* and *Corynebacteria* are among the most abundant bacterial species found on human skin. Bacteria belonging to these genera along with the Cutibacterium genus encompass between 45 and 80% of the entire human skin microbiome (17). These bacteria are primarily found in dry, moist, and sebaceous sites across the body (18), providing a large surface area to influence mosquito olfaction. *Anopheles* mosquitoes showed high attraction to the scent of diverse human skin bacteria, including *Corynebacterium* sp. (*19*). Additionally, out of the 15 volatiles collected from cultures of human foot microbes, five are also components of the volatile bouquet produced by *Staphylococcus epidermidis* (20). Both bacterial species have been profiled for the production of multiple carboxylic acids specific to the human skin that drive mosquito host-seeking behavior (21). *S. epidermidis* is one of 31 “core” members of the human skin microbiome (22) and, until recently, has been notoriously difficult to genetically engineer. L-Lactate dehydrogenase genes (*l-ldh*) have been identified in both *S. epidermidis* and *Corynebacterium amycolatum* genomes, indicating a potential to produce L-(+)-lactic acid during growth. We postulated an alternative approach to preventing mosquito bites is the genetic modification of the human skin bacteria to reduce or eliminate the secretion of mosquito attractive odorants (7, 8).

To validate the function of *l-ldh* gene of human skin bacteria *S. epidermidis* and *C. amycolatum* in generating L-(+)-lactic acid and attracting mosquitoes, we created *l-ldh* null mutants (Δ*l-ldh)* of both bacterial species. These engineered strains were then tested for attractiveness in a culture-based high-throughput olfactometer assay and in the context of mouse colonization for up to 14 days. We confirmed the critical role of *l-ldh* gene of *S. epidermidis* and *C. amycolatum* in their mosquito attraction in vitro and in vivo. Moreover, we validate the efficacy of these mutant human skin bacteria in reducing mosquito landing and biting using the two-choice noncontact assay and three-choice contact assay. Together, our findings demonstrate the importance of the *l-ldh* gene of human skin bacteria in augmenting the host-seeking process of mosquitoes and support the intriguing perspective of a “living” and long-lasting engineered microbiome-based mosquito repellent.

## Results

### Skin bacteria deficient in L-(+)-lactic acid production are less attractive to mosquitoes

To determine whether engineered skin bacteria could reduce mosquito attraction, we first developed strains of *S. epidermidis* and *C. amycolatum* that were deficient in L-(+)-lactic acid production. To do this, we generated L-lactate dehydrogenase gene deletions in *S. epidermidis* (Supplementary Fig. S1A, B) and *C. amycolatum* (Supplementary Fig. S1C, D), giving rise to *S. epidermidis* Δ*l-ldh* and *C. amycolatum* Δ*l-ldh*. Neither strain exhibited significant growth defects compared to the parental strain (Supplementary Fig. S2A, B). Importantly, both Δ*l-ldh* strains also demonstrated a significant reduction in L-lactate production (Supplementary Fig. S2C, D).

**Fig. 1.**
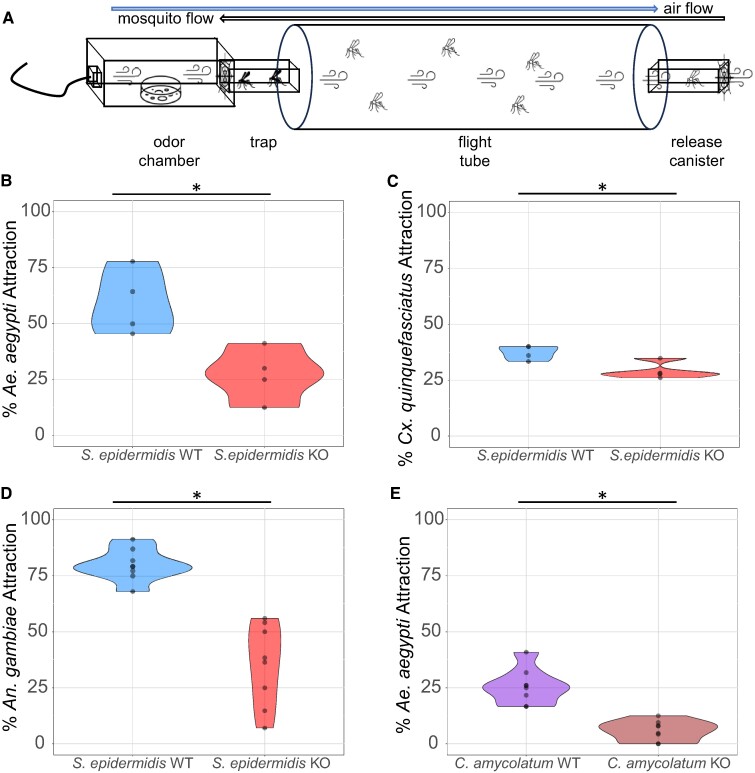
Mosquito attraction to the scent of human skin bacteria cultures in a Quattroport olfactometer. A) Schematic of one lane of the Quattroport olfactometer. Attraction to the scent of *S. epidermidis* WT and L-(+)-lactic acid knockout Δ*l-ldh* (KO) cultures by the mosquitoes *A. aegypti* (B), *C. quinquefasciatus* (C), and *A. gambiae* (D). E) Attraction of the *A. aegypti* to the scent of cultures of *C. amycolatum* WT and Δ*l-ldh* (KO). *n* = 4–8 biological replicates as represented by each dot. * *P* < 0.05.

As L-(+)-lactic acid, along with carbon dioxide, triggers mosquito short-range attraction (9, 14) and landing (21) behaviors, we implemented a Quattroport olfactometer (23) to assay the attractive/repellent potential of *S. epidermidis* and *C. amycolatum* and their respective Δ*l-ldh* counterparts (Fig. 1A). In these experiments, *S. epidermidis* Δ*l-ldh* exhibited reduced attraction to three genera of mosquito species including *Aedes aegypti* (54.2% reduced attraction, Fig. 1B), *Culex quinquefasciatus* (21.7% reduced attraction, Fig. 1C), and *Anopheles gambiae* (55.9% reduced attraction, Fig. 1D) as compared to wild type (WT). To further validate these findings, we assessed the attraction of the mosquito *A. aegypti* to *C. amycolatum* Δ*l-ldh* and showed that these mosquitoes were less attracted to the scent of Δ*l-ldh* as compared to WT cultures (77.1% reduced attraction, Fig. 1E). These results demonstrate that, as expected, cultures of the human skin commensals, *S. epidermidis* and *C. amycolatum*, are attractive to *A. aegypti*, *C. quinquefasciatus*, and *A. gambiae*. Furthermore, this attraction is significantly reduced when exposed to bacteria engineered to eliminate the production of L-(+)-lactic acid (Δ*l-ldh*).

### *S. epidermidis* deficient in L-(+)-lactic acid production makes mice less attractive to mosquitoes for multiple days

**Fig. 2.**
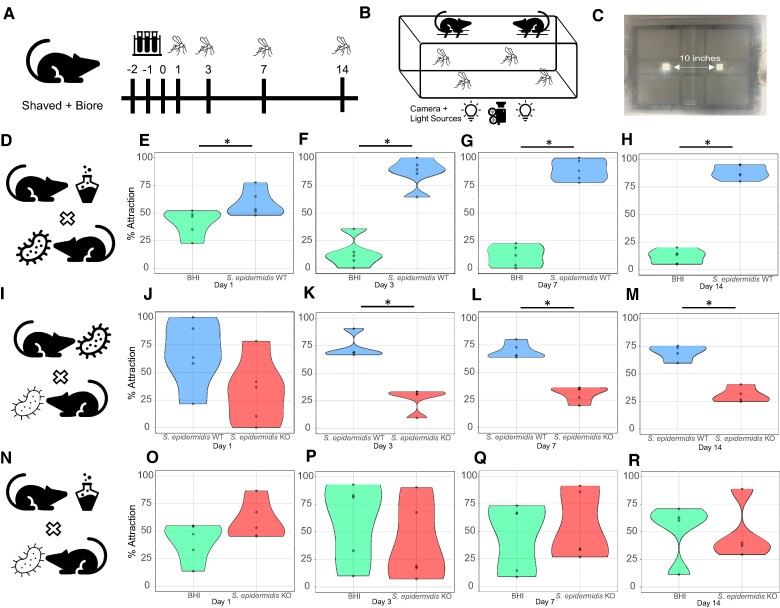
Attraction of the mosquito *A. aegypti* to the scent of mice treated with *S. epidermidis* WT strain, L-(+)-lactic acid knockout Δ*l-ldh* strain (KO), and culture media (BHI), in a two-choice noncontact assay. A) Schematic diagram of mouse skin treatment for skin bacteria engraftment and BHI media coating. B) Diagram of the two-choice behavioral arena. C) Picture depicting the lid of the behavioral arena, highlighting the two windows from which the mosquitoes sense the mice scent. Mosquito attraction to mice coated with D) BHI media or engrafted with *S. epidermidis* WT on days 1 (E), 3 (F), 7 (G), and 14 (H) after mouse skin treatment. Mosquito attraction to mice treated with either I) WT or Δ*l-ldh* (KO) strains on days 1 (J), 3 (K), 7 (L), and 14 (M) after mouse skin treatment. Mosquito attraction to mice treated with either N) BHI media or Δ*l-ldh* (KO) strain on days 1 (O), 3 (P), 7 (Q), and 14 (R) after mouse skin treatment. *n* = 4–5 biological replicates as represented by each dot. * *P* < 0.05.

Mosquito behavior in the context of all host cues (heat, CO_2_, breath, skin odors) is more complex than the strictly in vitro olfactometer testing behavior. Therefore, to capture these cues, we leveraged a two-choice noncontact behavioral assay (21) to compare mosquito attraction to mice colonized with *S. epidermidis* or *S. epidermidis* Δ*l-ldh* or mice coated with growth media [brain heart infusion (BHI)]*. S. epidermidis* cultures were applied onto the skin of mice for 3 consecutive days (Fig. 2A), and *A. aegypti* attraction to these mice was compared to mice treated with culture (BHI) media alone (Fig. 2B, C). Mice engrafted with *S. epidermidis* exhibited greater attraction to mosquitoes compared to BHI-treated mice (Fig. 2D) after 1 (30.5% attraction, Fig. 2E), 3 (84.3% attraction, Fig. 2F), 7 (86.9% attraction, Fig. 2G), and 14 (84.5% attraction, Fig. 2H) days of skin treatment. Further supporting the role of *S. epidermidis* in this increased attraction, *S. epidermidis* was detected at 1 and 14 days after treatment via PCR (Supplementary Fig. S3A, B).

We next assessed whether mice colonized with *S. epidermidis* Δ*l-ldh* led to reduced mosquito attraction compared to the WT strain (Fig. 2I). Apart from day 1 (Fig. 2J), *S. epidermidis* Δ*l-ldh-*treated mice showed reduced attraction on days 3 (64.4% reduced attraction, Fig. 2K), 7 (56.2% reduced attraction, Fig. 2L), and 14 (55.3% reduced attraction, Fig. 2M) after colonization as compared to mice colonized with the WT strain. *S. epidermidis* Δ*l-ldh* may produce D-(+)-lactic acid (*d-ldh*  **SERP2087**), however, as it contains a putative lactate racemase gene (ATM22_10565) that may convert D-lactate to L-lactate. To address the possible impact of this alternative L-lactate production pathway on mosquito attraction, we tested mosquito attraction to mice colonized with *S. epidermidis* Δ*l-ldh* and compared to mice treated with BHI media (Fig. 2N). Mosquitoes did not exhibit differential attraction toward *l-ldh* knockout treated mice at any time point (Fig. 2O–R), despite residual production of lactate by *S. epidermidis* Δ*l-ldh* (Supplementary Fig. S2). Together, these findings demonstrate that *S. epidermidis* Δ*l-ldh* abrogated mosquito attraction to mice for 11 uninterrupted days without any residual attractive effect.

### *C. amycolatum* deficient in L-(+)-lactic acid production makes mice less attractive to mosquitoes for multiple days

**Fig. 3.**
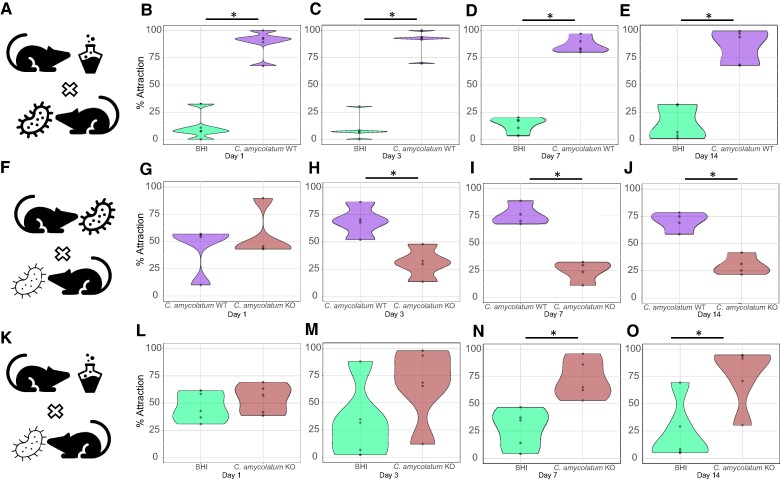
*A. aegypti* attraction to *C. amycolatum* WT strain, L-(+)-lactic acid knockout strain Δ*l-ldh* strain (KO), and culture media (BHI)-treated mice in a two-choice noncontact assay. Mosquito attraction to mice treated with BHI media or WT cultures (A) after 1 (B), 3 (C), 7 (D), and 14 (E) days. Mosquito attraction to mice treated with WT or Δ*l-ldh* (KO) cultures (F) after 1 (G), 3 (H), 7 (I), and 14 (J) days. Mosquito attraction to mice treated with BHI media or Δ*l-ldh* (KO) cultures (K) after 1 (L), 3 (M), 7 (N), and 14 (O) days. *n* = 4–5 biological replicates as represented by each dot. * *P* < 0.05.

*Corynebacterium* spp. also represents a significant proportion of the human skin microbiome and plays a role in mosquito attraction (19). Significant mosquito behavioral changes to mice colonized with *S. epidermidis* Δ*l-ldh* led us to consider whether this phenomenon is conserved in another prominent skin commensal, *C. amycolatum.* To this end, following experimental procedures used to test *S. epidermidis* colonized mice, we tested *C. amycolatum* WT or Δ*l-ldh*-colonized mice and assessed mosquito attraction in the two-choice behavioral model (Fig. 3).

As observed for *S. epidermidis*, *C. amycolatum* WT-colonized mice evoked much stronger mosquito attraction than BHI media-associated mice (Fig. 3A) on days 1 (91.8% attraction, Fig. 3B), 3 (92.3% attraction, Fig. 3C), 7 (79.7% attraction, Fig. 3D), and 14 (82.7% attraction, Fig. 3E) post-association. Next, we compared mosquito preferential attraction to mice treated with *C. amycolatum* Δ*l-ldh* and mice treated with the WT strain (Fig. 3F–J). Analogous to *S. epidermidis* Δ*l-ldh*-colonized mice, mice colonized with *C. amycolatum* Δ*l-ldh* showed reduced attraction to female *A. aegypti* mosquitoes when compared with WT-colonized mice (Fig. 3F–J) on days 3 (55.4% reduced attraction, Fig. 3H), 7 (68.0% reduced attraction, Fig. 3I), and 14 (57.4% reduced attraction, Fig. 3J) after skin engraftment. However, *C. amycolatum* still exhibited some attractive residual effects (Fig. 3K–O). In trials between mice treated with either BHI media or *C. amycolatum* Δ*l-ldh*, the latter group exhibited significantly greater attraction to *A. aegypti* on days 7 (62.2% attraction, Fig. 3N) and 14 (69.1% attraction Fig. 3O) after skin colonization. Despite exhibiting residual attractive effects, mice treated with *C. amycolatum* Δ*l-ldh* repelled mosquitoes compared to WT-colonized mice.

### Colonization with *S. epidermidis* Δ*l-ldh* reduces mosquito feeding propensity

**Fig. 4.**
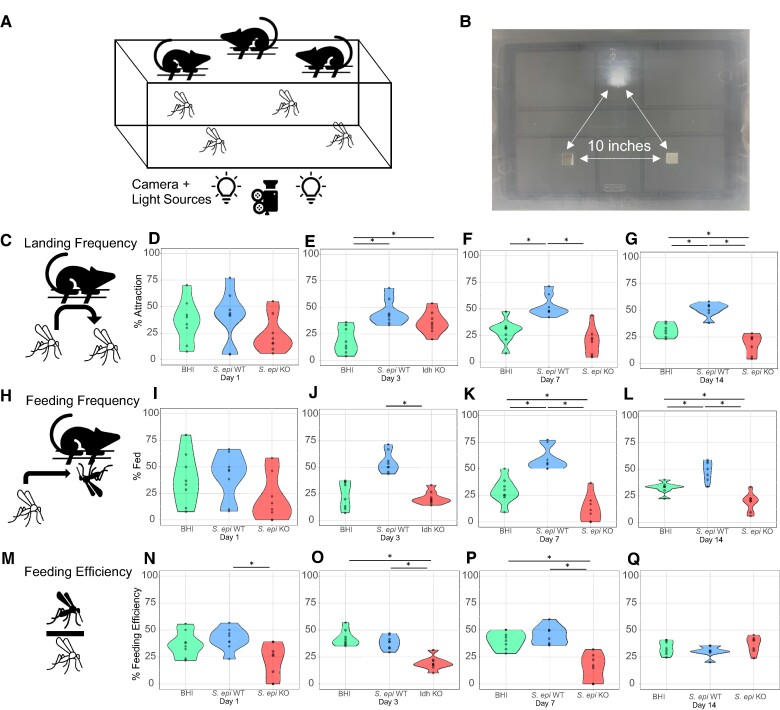
Attraction and feeding behavior of the mosquito *A. aegypti* exposed to mice treated with *S. epidermidis* WT strain, L-(+)-lactic acid knockout Δ*l-ldh* strain (KO), and culture media (BHI) in a three-choice contact assay. A) Diagram of the three-choice behavioral arena. B) Image depicting the lid of the behavioral arena, highlighting the three equidistant windows from which the mosquitoes sense the scent and feed upon the mice. Mosquito attraction (landing frequency, C) to mice on days 1 (D), 3 (E), 7 (F), and 14 (G) after mouse skin treatment. Mosquito feeding (feeding frequency, H) on mice after 1 (I), 3 (J), 7 (K), and 14 (L) days of skin treatment. Mosquito feeding efficiency (% # fed/# attracted, M) on days 1 (N), 3 (O), 7 (P), and 14 (Q) after mouse skin treatment. *n* = 7–8 biological replicates as represented by each dot. * *P*(adjusted) < 0.05.

To elucidate the effect of skin bacteria colonization on mosquito feeding behaviors, we modified the behavioral arena to a three-choice contact assay (Fig. 4A, B). In this assay, *A. aegypti* were exposed to mice treated with *S. epidermidis* WT, *S. epidermidis* Δ*l-ldh*, or BHI media and allowed to choose which one to feed upon. In this three-choice setup, mosquitoes still displayed reduced attraction (Fig. 4C–G) to mice treated with *S. epidermidis* Δ*l-ldh* compared to the WT strain on days 7 (64.2% reduced attraction, Fig. 4F) and 14 (64.6% reduced attraction, Fig. 4G) after skin engraftment. Even though mice treated with *S. epidermidis* Δ*l-ldh* presented greater attraction than BHI media-treated ones on day 3 (46.2% attraction, Fig. 4E), that residual attractive effect was lost on day 7 (Fig. 4F) and turned into reduced attraction on day 14 (42.1% reduced attraction, Fig. 4G).

Using this model, we assessed whether this *S. epidermidis* Δ*l-ldh* also affects *A. aegypti* feeding abilities (Fig. 4H–L). By determining the number of mosquitoes that engorged from each mouse treated with *S. epidermidis* WT, *S. epidermidis* Δ*l-ldh*, or BHI medium, we observed that fewer mosquitoes took blood meals from mice treated with the Δ*l-ldh* strain compared to WT-treated mice on days 3 (61.2% deterrence, Fig. 4J), 7 (80.6% deterrence, Fig. 4K), and 14 (60.7% deterrence, Fig. 4L). Furthermore, similar numbers of mosquitoes on days 1 and 3 (Fig. 4I–J) and fewer mosquitoes on days 7 and 14 (41.5–60.7% deterrence range, Fig. 4K–L) after colonization took blood meals from mice treated with the Δ*l-ldh* strain compared to BHI media-treated mice.

Besides reducing mosquito landing and feeding for multiple days, it is important to assess whether mice treated with *S. epidermidis* Δ*l-ldh* strain affect mosquito feeding efficiency (Fig. 4M–Q): the ratio of the number of mosquitoes that actually fed upon each mouse out of the mosquitoes that landed on them (Fig. 4M). Feeding efficiencies were reduced upon mice colonized with *S. epidermidis* Δ*l-ldh* when compared to the WT treated on days 1 (48.5% reduced feeding efficiency, Fig. 4N), 3 (49.7% reduced feeding efficiency, Fig. 4O), and 7 (64.7% reduced feeding efficiency, Fig. 4P) after skin treatment. Mice treated with *S. epidermidis* Δ*l-ldh* displayed reduced feeding efficiency to mosquitoes on days 3 (54.4% reduced feeding efficiency, Fig. 4O) and 7 (61.1% reduced feeding efficiency, Fig. 4P) compared to mice associated with BHI media. Altogether, these findings indicate that *S. epidermidis* Δ*l-ldh* evokes a feeding deterrent behavior in mosquitoes that lasts at least 1 week.

## Discussion

Mosquito host-seeking behavior is mediated by the volatiles released by the human breath and by the resident skin bacteria (7). Among those, carbon dioxide mediates mosquito activation and hierarchically interacts with L-(+)-lactic acid, ammonia, and other short- and middle-chain carboxylic acids to induce strong host attraction (10, 11, 14, 24). The absence of components such as L-(+)-lactic acid and ammonia in an odor blend significantly reduces the ability of mosquitoes to perform odor-mediated attraction and landing (9–11, 21).

DEET remains the gold standard in topically applied repellents (> 90% reduction in attraction)(3). However, the need for constant re-application [within hours (4)] leads to logistical issues and is cost prohibitive in malaria endemic regions worldwide. Among novel strategies to prevent mosquito bites, the reengineering of the human skin microbiome to produce repellents and/or reduced levels of attractive odorants may be realized as a more stable and long-lasting solution (7, 25). As L-(+)-lactic acid and ammonia are key odorants to gate mosquito human-seeking behavior, reducing the production of such odorants by the skin microbiome might result in an effective strategy to reduce mosquito bites and pathogen transmission.

In this study, we engineered two common human skin commensals, *S. epidermidis* and *C. amycolatum*, and significantly decreased their production of L-(+)-lactic acid through deletion of the L-lactate dehydrogenase (Δ*l-ldh*) gene. We also demonstrated that mosquitoes are less attracted to the scent of the Δ*l-ldh* strains than to WT in vitro. These findings prove that skin bacterium deprived of the ability to produce L-(+)-lactic acid is key to reducing mosquito attraction, despite these bacteria producing other human skin-derived odorants (20).

Building upon these findings, we tested the effects of these strains on mosquito behavior after colonizing a mouse model in two-choice noncontact or three-choice contact assays. Upon engraftment, WT strains increased mosquito attraction for 2 weeks. In contrast, mice colonized with Δ*l-ldh* counterparts are less attractive to mosquitoes for 11 consecutive days with little to no residual attractive effect. Mosquito feeding desire is also reduced for 7 consecutive days. While not equivalent to protection conferred by DEET or picaridin (90–100%) (3), we observed significant reduced attraction (55.3–68%) and deterrence (60.7–80.6%) mediated by Δ*l-ldh* skin bacteria. Notably, this protection lasted 7–11 days post-application, whereas DEET/picaridin is only effective for a few hours, requiring constant re-application (3, 4).

The human skin microbiome is very stable over time [months and years (26, 27)]. The individual genetics rather than the environment seems to define the composition of the skin microbiome (27). Although skin microbiome variability due to ethnicity and gender is not insignificant (17), these factors appear to be secondary and are associated with hygiene, which apply only to the microbiome on the skin surface (17). The microbiome of the deep skin layers is more universal (28) and responsible for recolonizing the skin upon tissue skin injury (29).

This study demonstrates the potent effect of human skin commensal-derived L-(+)-lactic acid on mosquito attraction and feeding efficiency for 7–11 days. As an approach, living mosquito repellents benefit from (i) durable, self-replicating protection (no lapse in protection concerns), (ii) low logistical burden, and (iii) significantly cheaper lifetime protection. Even though the Δ*l-ldh* strains display similar growth curves in culture to WT and could be detected on the mouse skin 14 days after engraftment, it is still an open question whether or not such L-(+)-lactic acid-deficient strains can colonize the human skin and compete against the WT microbiome. Provided genetically engineered (Δ*l-ldh)* skin bacteria can grant effective and long-lasting reduced attraction to the human skin, this novel strategy could be used alone or in combination with topical application of synthetic repellents to reduce vectorial capacity and provide long-lasting skin protection from mosquito bites, pathogen transmission, and mosquito-borne diseases.

## Materials and methods

### Mosquito rearing

*A. aegypti* Liverpool, *C. quinquefasciatus* WT S-strain, and *A. gambiae* G3 strain mosquitoes were raised at 28.0 °C and 70% relative humidity (12-hour light/dark cycle). Larvae were fed with ground fish food (TetraMin Tropical Flakes, Tetra Werke, Melle, Germany), and the *A. gambiae* diet was supplemented with 2% beef liver powder (NOW, Bloomingdale, IL, USA). Adults were provided 0.3 M aqueous sucrose *ad libitum*. Adult females were blood fed 3 to 5 days after emergence upon anesthetized mice. All animals were handled in accordance with the Guide for the Care and Use of Laboratory Animals as recommended by the National Institutes of Health and supervised by the local Institutional Animal Care and Use Committee (S17187) and the Animal Care and Use Review Office (ACURO) protocol DARPA-9729.

### Genetic engineering of skin bacteria

*S. epidermidis* NIHLM087 (ATM22_01530) was obtained from the NIH (30). *C. amycolatum* ATCC 49368 (16165_RS07200) was purchased from ATCC: The Global Resource Center. All bacterial strains were grown in Difco BHI media (BD 237200) at 37 °C with shaking in which *Corynebacterium* species were cultured in BHI media supplemented with 1% Tween-80 (BHIT). Prior to liquid growth, individual colonies were cultured overnight on respective BHI (for *Staphylococcus* strains) or BHIT (for *Corynebacterium* strains) agar plates. To delete *l-ldh* genes in *S. epidermidis* and *C. amycolatum*, ∼1,000 bp directly upstream and downstream of the *l-ldh* gene were amplified for each strain and the PCR products were cloned into the pIMAY (for *S. epidermidis* NIHLM087; Addgene Plasmid #68939) and pJSC232 (for *C. amycolatum* ATCC 49368) temperature-sensitive vectors using Gibson assembly. Transformation of the species-specific deletion vector was performed as described in (31). To generate electrocompetent cells, overnight *S. epidermidis* grown in BHI supplemented with 0.5 M sorbitol (Sigma) (BHIS) and *C. amycolatum* grown in BHIT supplemented with 0.5 M sorbitol (BHIST) were back-diluted to an optical density of 0.15 for *S. epidermidis* and 0.3 for *C. amycolatum*. Back-diluted cultures were placed on ice once they reached an OD of 0.7 for *S. epidermidis* and 1.2 for *C. amycolatum* and pelleted at 3,500 *g* for 10 minutes at 4 °C. Spun-down cultures were resuspended in equal volume with 10% ice-cold glycerol followed by five 10% glycerol washes. After the last wash, cells were suspended in 100 µL of 10% ice-cold glycerol to use for electroporation.

Approximately 1 µg of DNA of plasmid isolated from DC10B and TOP10 *Escherichia coli* was added, respectively, to 100 µL of competent *S. epidermidis* and *C. amycolatum*. For methods specific to *S. epidermidis*, cells and plasmids in 10% glycerol were first heat-shocked at 56 °C for 2 minutes followed by immediate electroporation in 0.1-cm cuvette (Bio-Rad) using electroporation program of 2.5 kV and time constant of 2.3–2.5 ms on the Bio-Rad Micropulser. Electroporated cells were then transferred to 3 mL of prewarmed BHIS and recovered at 37 °C for 3 hours prior to plating on BHIS plates with appropriate antibiotics. For methods specific to *C. amycolatum*, plasmid was electroporated into competent cells in 0.2-cm cuvette with 2.5 kV and time constant of 4 ms and immediately heat-shocked in BHIST that was previously prewarmed at 46 °C for 6 minutes and then recovered at 37 °C for 3 hours prior to plating on BHIST plates with appropriate antibiotics.

To create targeted deletion of *l-ldh* in *S. epidermidis* LM087 and *C. amycolatum* ATCC 49368 (31), once species-specific deletion plasmid was electroporated into each strain, transformed cells were selected on 10 µg/mL chloramphenicol (for *S. epidermidis*) and 25 µg/mL kanamycin (for *C. amycolatum*) for a single chromosomal crossover, followed by selection solid medium containing 1 µg/mL anhydrotetracycline (for *S. epidermidis*) and 10 µg/mL sucrose (for *C. amycolatum*), which produces either complete gene deletions or WT bacteria revertants. Gene deletions are verified by PCR and Sanger sequencing.

All the primers used in the above experiments are listed in Supplemental Table S1. *S. epidermidis* LM087 Δ*l-ldh* was fully sequenced (GenBank accession number: PRJNA1129207) on an Illumina NextSeq500 and compared to Wt LM087 using Snippy (https://github.com/tseemann/snippy) and found to have 100% coverage with no SNPs or indels, except for the absence of 855 bp found within the L-ldh gene.

### Quattroport olfactometer assay

Mosquito behavioral assays were performed with a modified Quattroport high-throughput olfactometer (23) at 27 °C and 80% relative humidity. The screens of the olfactometer's traps were removed to allow mosquitoes to make a choice between staying close or moving far away from the odor source. Twenty female mosquitoes were transferred to the releasing canisters and starved for 5–8 hours without water. Purified air was pumped into the system at 24,367 mL/min rate, whereas pure CO_2_ was flown at 254 mL/min (final concentration per lane ∼1,500–2,000 ppm). Mosquitoes were exposed to air for 5 minutes, when bacterial cultures (1,000 μl) were placed in the odor chamber onto 47-mm plastic petri dishes (Fisher Scientific, Hampton, NH, USA), and CO_2_ gauge was switched on. The gates of the releasing canisters were open, and the behavioral assays were carried out for 20 minutes. Then, both the releasing canister and the trap gates were closed, and the number of mosquitoes in the releasing canisters, flight tubes, and traps was scored.

### Mouse engraftment procedure

WT and Δ*l-ldh S. epidermidis* and *C. amycolatum* strains were inoculated onto BHI plates from frozen stocks. Single colonies were picked to individually inoculate 4 mL of BHI media in 15-mL culture vials for overnight growth before engraftment. OD_600_ values of WT and Δ*l-ldh* cultures were measured with Nanodrop and normalized to 2.0 with BHI media. For skin engraftment, 6-to-8-wk-old female C57BL/6 mice were purchased from Jackson Laboratory (Jax). Twenty anesthetized mice were shaved in either the abdomen or flank region. Native microbes were removed using Biore Deep Cleansing Pore Strips (Biore, Cincinnati, OH, USA) following the manufacturer's instruction (10-minute application). Bacterial strains were engrafted on the shaved abdomen of mice by dipping a swab (ESwab Collection Kit, Becton, Dickinson, and Company, Franklin Lakes, NJ, USA) into the bacterium culture and swabbing the exposed skin of the mouse abdomen 15 times for three consecutive days. The same procedure was conducted with bacterial BHI medium, which served as a control for the experiment.

### Two-choice noncontact mouse assay

Plastic containers (50 cm × 30 cm × 15 cm, Hefty) were modified by cutting two square windows (1 × 1 inch) on the lid 30 cm (10 inches) apart. Square polyester meshes (2 × 2 inches) were used to cover the windows from the inner side to prevent mosquito escape. Custom-made plastic frames [1/16″ thick, gates used for the Quattroport olfactometer (23)] with small holes were placed on the outer side of the windows to create a short distance between mouse skin and mosquito, preventing the mosquitoes from having physical contact with the mice. Twenty mosquitoes were introduced into the box using a mouth aspirator (John Hock, Gainesville, FL, USA) before the trials. Two mice (BHI-treated versus WT bacteria-treated, WT bacteria-treated versus Δ*l-ldh* bacteria-treated, or BHI-treated versus Δl-*ldh* bacterium-treated) were placed on the top of plastic frames with their shaved abdomen facing the experimental cage. Host-seeking activity of mosquitoes was recorded for 10 minutes. Mice with different treatments were switched between windows across trials to prevent positional bias. The videos were processed by manually counting the landing frequency of mosquitoes.

### Three-choice contact mouse assay

The lids of similar arenas used in the two-choice assays were modified by cutting three square windows (1 × 1 inch) 10 inches apart in a triangular shape. Unlike the two-choice noncontact assay, no mesh was used to cover the window, and no plastic frame was placed on top of the window, enabling the mosquito to contact the mouse skin and initiate blood feeding. Twenty female mosquitoes were introduced into the arena with a mouth aspirator. Three mice treated with BHI media, WT *S. epidermidis*, or Δ*l-ldh S. epidermidis* were placed on the windows with their shaved abdomen facing the arena. The host-seeking and blood feeding activities of mosquitoes were recorded for 10 minutes. To eliminate any potential position effect, we interchanged the position of the mice with different treatments across replicates. The videos were further processed by manually counting the mosquito landing and feeding.

### Video recording of behavioral activity

For the two- and three-choice assays, videos of mosquito activity were recorded with an iPhone X at 30 fps. Videos were analyzed by blinded, visual counting.

### Behavior apparatus cleaning

The Quattroport olfactometer parts were soaked overnight (small parts) or washed thoroughly (flight tubes) with scent-free laundry detergent (Seventh Generation Free and Clear) and rinsed with tap water thoroughly. For the two- and three-choice arenas, all the parts in contact with the mice were washed with the same detergent.

### Statistical analyses

Graphs and statistical analyses were performed with the R software using the ggplot2 package. For all experiments, the number of mosquitoes landing and feeding on each mouse and the number of mosquitoes caught by the traps were transformed into percentages to normalize mosquito participation variability across experimental replicates (Supplemental Table S1). Percent attraction was calculated as (1 − number of landing events on control mouse/number of landing events on treated mouse)×100. Percent reduced attraction was calculated as (1 − number landing events on treated mouse/number of landing events of control mouse)×100. Percent deterrence was calculated as (1 − number feeding events on treated mouse/number of landing events of control mouse)×100. Shapiro–Wilk normality test was used to assess whether the data fit a normal distribution. For pairwise comparisons, either the Welsh *t*-test or Wilcoxon rank sum test was used. For multiple comparisons, either ANOVA or Kruskal–Wallis's rank sum test was applied. These tests were followed by post hoc analyses using Tukey’s multiple comparisons of means and Wilcoxon’s rank sum test, respectively. Where indicated, *P*-values were adjusted (*P*-adjusted) for multiple comparisons using the Benjamini–Hochberg procedure. The raw data and R codes are provided in Supplemental Table S1.

## Supplementary Material

pgae267_Supplementary_Data

## Data Availability

Raw data used for each figure and R code are available in Supplemental Table S1. Genomic sequencing file is available under the GenBank accession number PRJNA1129207.

## Preprints

This manuscript was posted on a preprint: https://doi.org/10.1101/2023.12.20.572663.
