# Supplementary figures and images for: Engineered skin microbiome reduces mosquito attraction to mice
Source: PNAS Nexus. 2024 Jul 30;3(7):pgae267. doi: 10.1093/pnasnexus/pgae267 (PMC11287867; doi:10.1093/pnasnexus/pgae267)

A

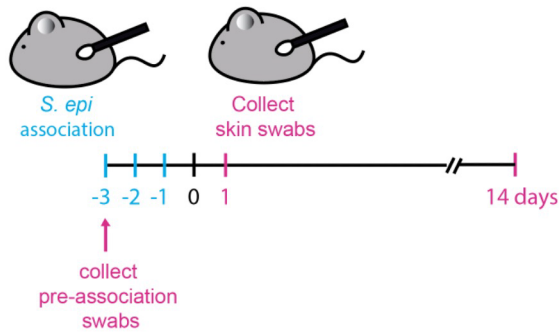

B

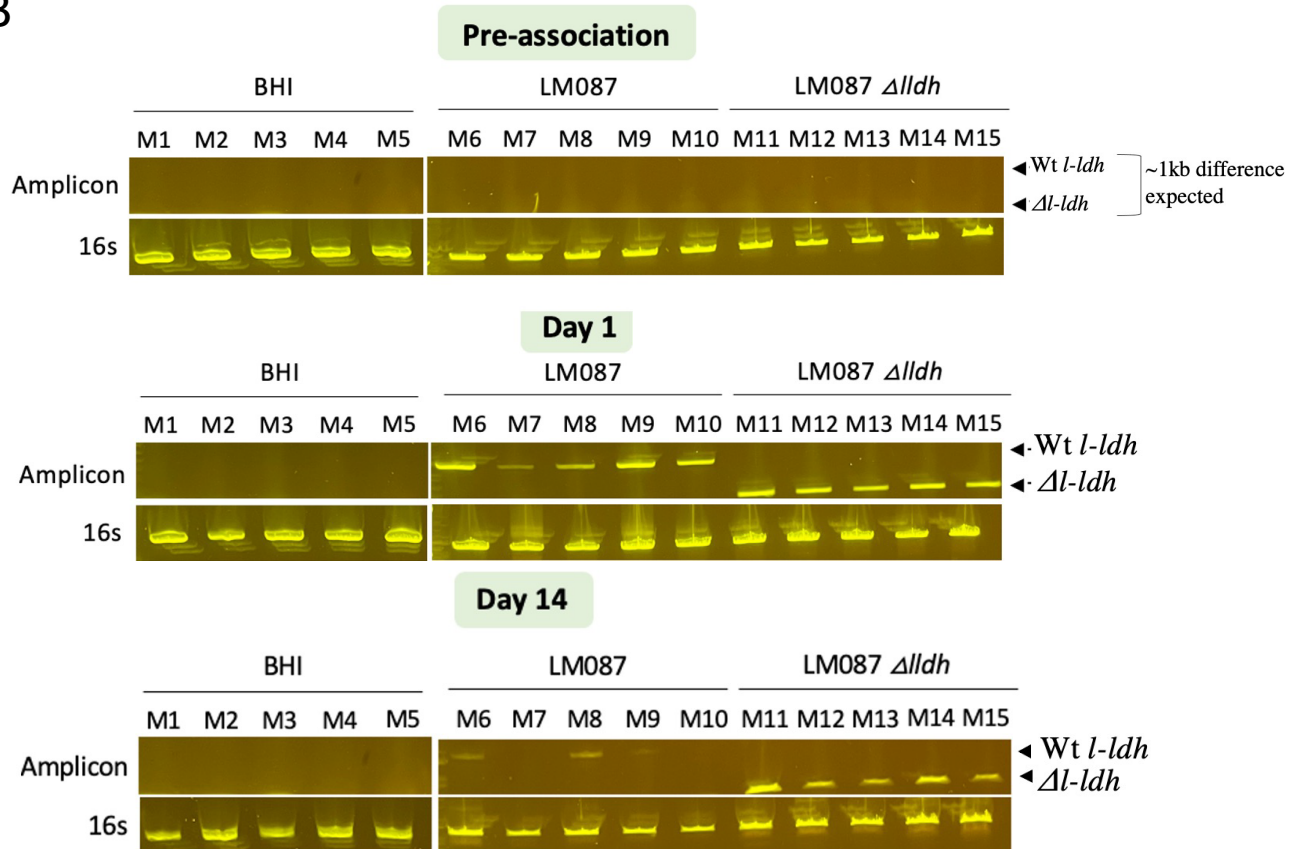

Supplement: pgae267_Supplementary_Data [file pgae267_supplementary_data.zip › PNASNEXUS-PNASNEXUS-2024-00219-TR-s01.pdf]

A

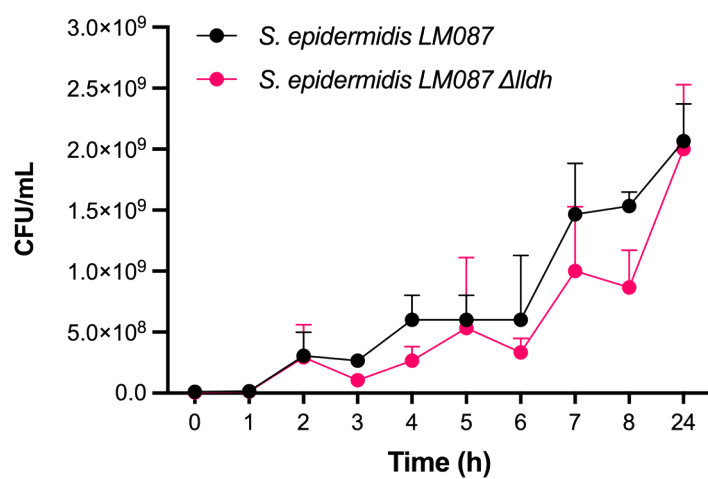

B

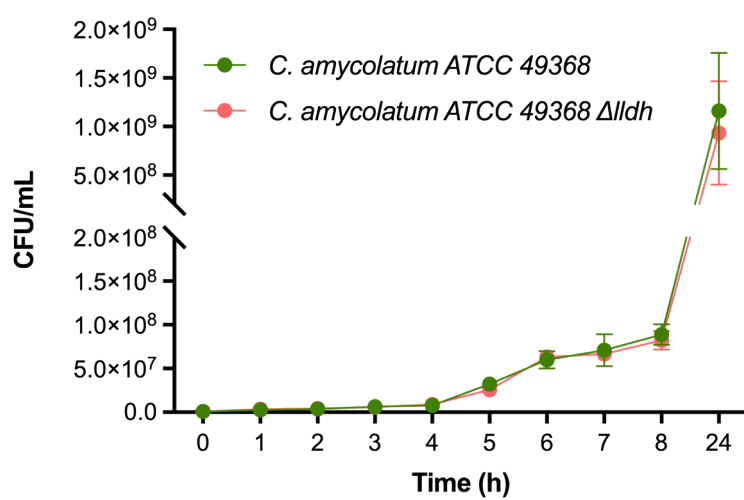

C

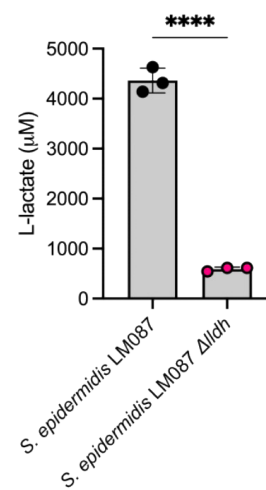

D

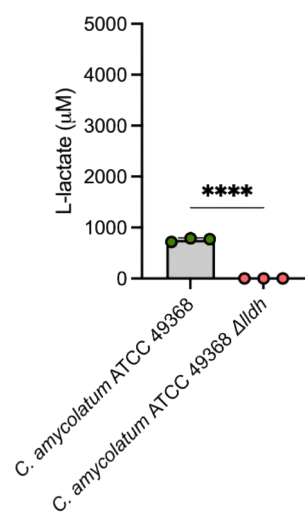

Supplement: pgae267_Supplementary_Data [file pgae267_supplementary_data.zip › PNASNEXUS-PNASNEXUS-2024-00219-TR-s02.pdf]

A

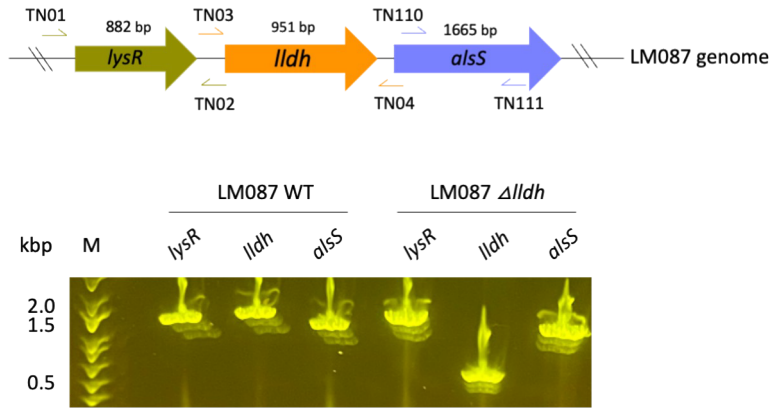

B

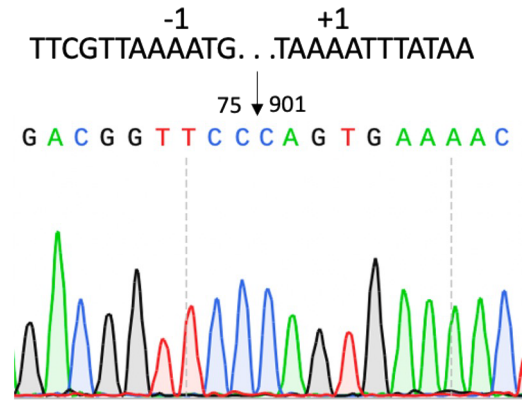

C

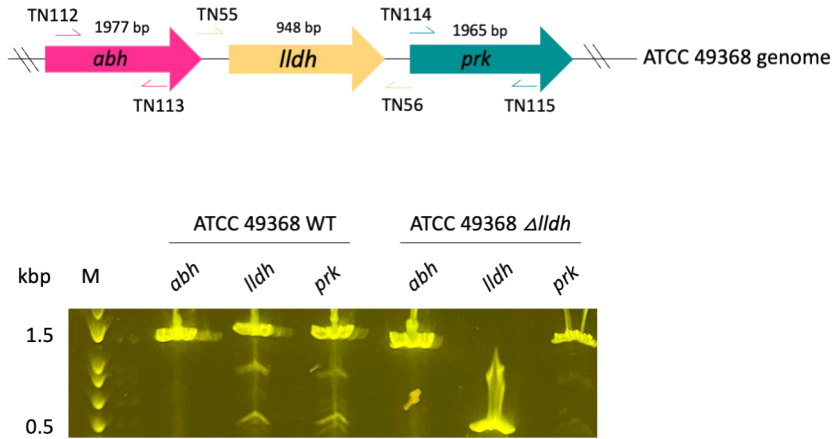

D

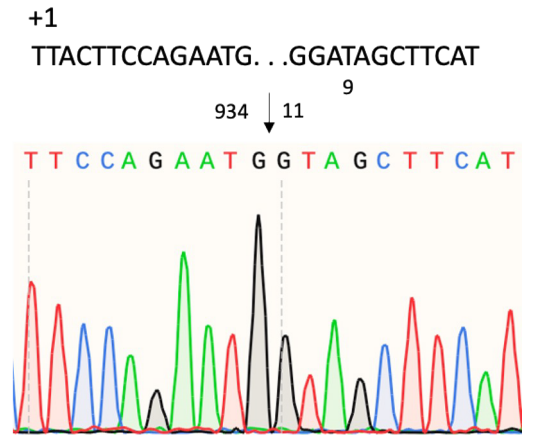

Supplement: pgae267_Supplementary_Data [file pgae267_supplementary_data.zip › PNASNEXUS-PNASNEXUS-2024-00219-TR-s03.pdf]
